# Model organism databases: essential resources that need the support of both funders and users

**DOI:** 10.1186/s12915-016-0276-z

**Published:** 2016-06-22

**Authors:** Stephen G. Oliver, Antonia Lock, Midori A. Harris, Paul Nurse, Valerie Wood

**Affiliations:** Cambridge Systems Biology Centre & Department of Biochemistry, University of Cambridge, Sanger Building, 80 Tennis Court Road, Cambridge, CB2 1GA UK; Department of Genetics, Evolution and Environment, and UCL Institute of Healthy Ageing, University College London, Darwin Building, Gower Street, London, WC1E 6BT UK; The Francis Crick Institute, 215 Euston Road, London, NW1 2BE UK

## Abstract

Modern biomedical research depends critically on access to databases that house and disseminate genetic, genomic, molecular, and cell biological knowledge. Even as the explosion of available genome sequences and associated genome-scale data continues apace, the sustainability of professionally maintained biological databases is under threat due to policy changes by major funding agencies. Here, we focus on model organism databases to demonstrate the myriad ways in which biological databases not only act as repositories but actively facilitate advances in research. We present data that show that reducing financial support to model organism databases could prove to be not just scientifically, but also economically, unsound.

Most of our knowledge about the basic properties of metabolism, growth, and division in living cells is a result of studies on species described as “model organisms”. These species include the bacterium *Escherichia coli*, bakers’ yeast (*Saccharomyces cerevisiae*), the fruit fly (*Drosophila melanogaster*), the nematode worm (*Caenorhabditis elegans*), the mouse (*Mus musculus*), and the thale cress (*Arabidopsis thaliana*). Model organism databases (MODs) host the genomic and functional information produced by organism-specific research projects and provide query and visualization tools to access these data. A recent commentary in *Science* [1] revealed that NIH institutes, notably including the NHGRI, propose to eliminate database funding, estimated at 0.37 % of the biomedical research budget, over the next 4 years as a cost-saving measure. Furthermore, funders suggest that merging databases might improve cost efficiency or that databases should introduce subscription-based funding. We contend that research on model species will continue to elucidate fundamental mechanisms that operate in most other species, including humans.

At every stage of the scientific process, MODs contribute to basic and applied research. By consulting MODs, researchers can easily find background information on large sets of genes, such as those involved in a biological process or implicated in a disease. MOD users can thus plan experiments efficiently, combine their data with existing knowledge, and construct novel hypotheses. Although the central role of MODs in research planning is seldom acknowledged in the literature, it sometimes is (e.g., for research on neuronal ceroid lupofuscinosis [2]) and it is well attested by personal communications to MOD staff. The typical use of PomBase in experimental planning, hypothesis generation, and data mining are also described in a recent *Genetics* Primer on fission yeast [3]. By gathering and interconnecting diverse types of information from many sources, MODs enhance the communication of knowledge gained via model organism-based research to the broader community. Moreover, the aggregated knowledge for a model species is now routinely used to perform sophisticated analyses that facilitate the interpretation of results [4–7].

We draw on our experience with MODs, especially the fission yeast database PomBase [8], to illustrate the changing nature of biological research and the ways biological databases adapt in response. We argue that emerging research developments increase, not decrease, the relevance and value of MODs as essential components of the global research infrastructure. Funding MODs and other biological databases is therefore already an efficient way to support biomedical research and MODs are exploring ways to increase their efficiency by streamlining database maintenance.

## Publication trends: the unbearable richness of data?

The quantity of literature produced using a species is one metric for the “success” of a model organism research community. In a recent Perspectives article, Dietrich et al. [9] assessed publication trends and showed that a “model organism” designation did not necessarily correlate to an increase in the number of publications. Indeed, they showed that fission yeast, *Neurospora*, *Dictyostelium*, and even bakers’ yeast had relatively constant rates of publication. We can confirm that the rate of publication for fission yeast is relatively stable at ~500 publications per year.

**Fig. 1.**
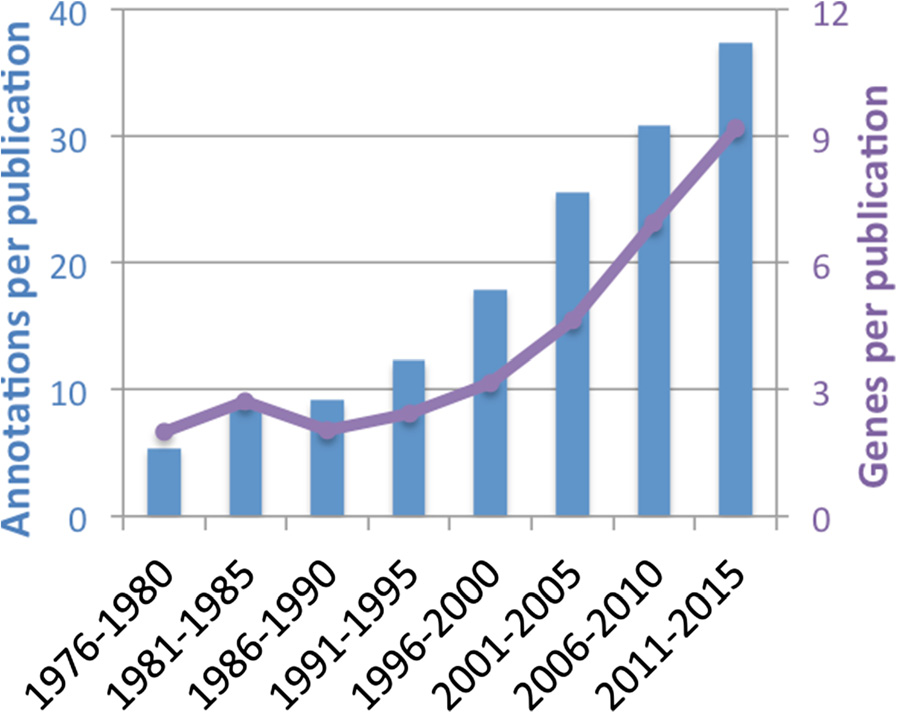
Mean number of manually curated annotations (*bars*) and genes annotated (*line*) in PomBase per peer-reviewed paper in 5-year intervals. Counts exclude high-throughput experiments and use the same criteria for all years

Closer inspection of curated data at PomBase, however, shows that raw paper counts obscure dramatic changes in publication content that reflect ongoing trends in basic research. Biocurators at each MOD organize the knowledge presented in the literature into building block-like units known as “annotations”, which connect genes to defined terms drawn from shared, structured, controlled vocabularies. These vocabularies, known as ontologies, describe attributes such as functions, phenotypes, modifications, etc. in a computation-compatible manner. Annotation numbers from PomBase illustrate that the information content per publication is increasing, even as publication numbers remain constant. From the mid-1970s until the mid-1990s, a typical publication might describe the cloning of a single gene and report a small amount of functional data. Figure 1 shows that about five to ten annotations were typically made per paper published during this period. This number increased steadily to over 20 gene annotations per publication by 2005 as individual laboratories applied a larger range of experimental techniques. In the past 5 years, even for small-scale experiments, the average number of gene annotations per paper has grown to 35 and the average number of genes studied has increased to almost 10. A typical publication in 2016 will frequently contain enough functional data to allow detailed models of novel parts of conserved cellular processes to be proposed [10, 11], or even report in vitro assays involving over 100 gene products [12]. Laboratories dedicated to systems-wide approaches will typically identify even larger sets of genes based on experimental perturbations of a biological system of interest. While they were initially applied in purely exploratory studies, high-throughput methods are now used to extend hypothesis-driven research to greater numbers of genes and experiments [13]. To interpret system-scale results, researchers identify common features of gene sets based on the consistent knowledge framework provided by the relevant MOD [3–7, 12]. Clearly, it would be impossibly time-consuming for individual laboratories to compile and maintain the catalogues of gene-specific information required to be productive in the modern research environment.

## Reuse of curated data: where *do* all the other annotations come from?

In addition to fulfilling their original mandate to support specific model species, the experimental annotations that MODs create yield benefits that reach far beyond model organism research communities. With over 15,000 genome sequences now completed or in progress [14], the potential uses of MOD annotations have expanded tremendously in recent years. For example, in the fungal kingdom alone, complete genomes are available for over 500 species. Many represent serious animal and plant pathogens, which threaten biodiversity and lead to global agricultural losses estimated at $60 billion per year [15]. The genus *Cryptococcus* alone causes over one million life-threatening human infections per year worldwide [16].

**Fig. 2.**
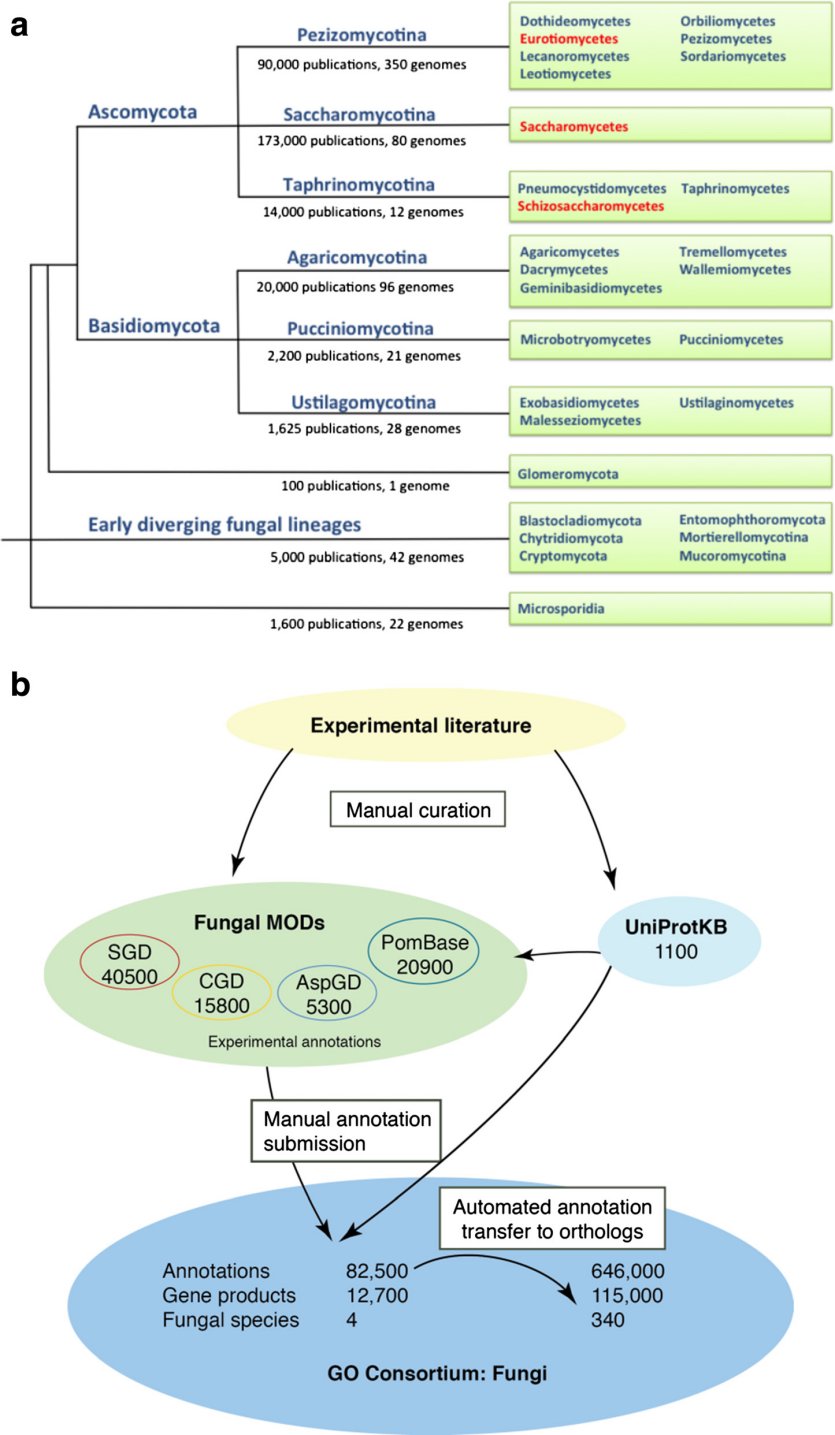
Sequenced fungal genomes and their functional annotation. **a** Phylogenetic tree showing selected fungal taxa in which one or more species has a genome sequence available. For subphyla (e.g., Pezizomycotina), the number of publications on species within the subphylum and the number of available genome sequences are shown. *Red text* denotes classes that include species for which a MOD exists. **b** Information flow in Gene Ontology annotation. Curators at MODs and UniProtKB create manual annotations based on published experiments; the number of such annotations is shown for each database. Some UniProtKB annotations are incorporated into MOD datasets and all are submitted to the GO repository. Annotations are then transferred to orthologous genes

Some non-model fungal species have already accumulated a substantial volume of literature; in other cases, entire fungal classes have little experimental data available. In neither case is there a dedicated effort to annotate the entire genome and proteome of these species or to curate their literature corpus (Fig. 2a). Often, the goals of preventing and treating fungal diseases direct research towards understanding pathogen–host interactions. Although the core cellular processes of pathogenic fungi are seldom studied directly in this context, successful identification of potential drug targets or fungicides nevertheless requires functional annotation of their basic conserved gene sets. MODs capably address this problem in that robust methods exist to partially automate annotation of gene function across all sequenced genomes. For example, Gene Ontology (GO) annotations can be accurately transferred from genes in one species to those in another based on sequence orthology and phylogenetic relationships [17, 18].

Figure 2b shows that fungal MOD data underpin hundreds of thousands of annotations generated for other fungi by automated methods. For basic cellular processes, annotations can even be transferred between kingdoms using phylogenetics and suitably stringent rules [18]. Despite the successes, it is inescapably true that accurate and informative gene-specific functional annotations can only be derived automatically from a foundation of annotations that have been curated manually from experiments reported in the literature. For the more than 500 sequenced fungal species, all of the gene-specific data available for propagation are provided by the experimentally derived annotations submitted to the GO database by the Saccharomyces Genome Database (SGD) [19], PomBase [9], UniProt [20], and others. As annotations are similarly transferred within (and between) other kingdoms, MOD annotation efforts provide the majority of the experimentally derived GO annotations from which the >275 million electronically inferred GO term assignments are subsequently derived and are therefore critical to sustain biological research on organisms from all branches of the tree of life.

## Sustainability

Biological database providers recognize that funding cannot increase linearly with data production, even under optimal circumstances. That said, funding restrictions, either implemented or imminent, further intensify the challenges that databases face in meeting increasing data demands. We note critical limitations of proposed cost-cutting measures such as subscriptions, automated curation, and resource mergers and we offer alternative suggestions for improving MOD productivity.

With the increasing use of cross-species comparisons in both experimentation and data analysis, researchers typically need to use several different databases, both organism-specific and general. For example, PomBase serves five times as many monthly unique visitors as the estimated total number of fission yeast researchers. Moving databases to subscription models is likely to result in uneven and fragmented access to different databases and risks greatly exacerbating existing disadvantages faced by less well-funded institutions and research groups. Implementing paid access will often just result in funders paying for the databases by an indirect route and one that incurs administrative overheads that divert money and effort away from curation.

We have shown that even partially automated annotation depends upon a high-quality reference set from which annotations can be transferred. Furthermore, there are currently no text-mining tools or other automated methods that can supply even a small fraction of the annotation detail captured by manual curation. MODs also curate data types that are far less amenable to orthology- or phylogeny-based transfer, such as phenotypes and targets of modifications. Automation cannot, therefore, entirely replace manual curation; instead, it is an increasingly important means of exploiting manual annotation datasets.

Although MODs have broadly similar goals, their diverse user communities have different requirements, legacy data, and future needs that pose practical limitations to outright merger. More importantly, any merged entity would still face the same curation workload as before. Instead of merging, MODs work together on a daily basis to share vocabularies and curation protocols [21, 22] as well as database schemas (e.g., Chado) and other tools (see the GMOD collection) [23, 24]. MODs continue to explore methods to improve efficiency by developing shared curation protocols and streamlining curation workflows.

One promising mechanism to increase sustainability is to harness the expert knowledge of database users in the curation effort. In 2013, PomBase launched a community curation project following a successful pilot and the development of a user-friendly community curation tool, Canto [25]. To date, over 100 fission yeast laboratories have participated, curating >300 publications to provide >3300 annotations using defined ontology terms. Each new paper is effectively co-curated by a biological expert and a professional curator, a procedure that combines the topic-specific knowledge of the former with the latter’s familiarity with ontologies and annotation practices. This generates accurate, consistent, and highly specific annotations. Researchers who participate in community curation gain a better understanding of how their results are represented in the MOD, which in turn supports a positive feedback loop that improves curation accuracy and data usage. Other MODs have also had success with community curation, providing various levels of annotation [26, 27]. To increase the uptake and influence of community curation, we recommend that journals make participation a prerequisite for publication or funders include it as a data-sharing requirement (or, of course, both). In either case, curation can be attributed to individuals via ORCIDs, the recently devised unique digital identifiers for researchers (http://orcid.org/). Furthermore, publishers and funders could enforce the preparation of machine-readable abstracts and stricter adherence to minimal information standards to reduce the load on manual curation.

## Conclusions

We have illustrated that major changes are taking place in the research approaches used to study model species, as reflected in the content of the resulting publications. We contend that these emerging research trends can be attributed, in part, to the availability of an actively maintained MOD. We also describe one major way in which the curated data are subsequently reused to annotate many thousands of sequenced genomes; this has become so much the norm that the MOD from which annotations were transferred is rarely acknowledged. The activities of every MOD support their target research communities by providing essential access to information that no individual laboratory could amass unaided. Using PomBase as an example, we have described some aspects of the unrecognized role of MODs in supporting experimental design, hypothesis generation, and seeding annotation across diverse species.

Although individual MODs each have unique characteristics and responsibilities, we believe that our conclusions apply to all MODs. Some distinctive features of MODs include the fact many contain data for more than one species (e.g., WormBase [28] originally focused on just *C. elegans* but now hosts genomic data for ten *Caenorhabditis* species and a number of nematodes from other genera). Additionally, the number of relevant papers varies by more than 100-fold between the MODs and this requires different approaches to manual curation of the data these papers contain. Curating a large body of literature requires some form of “triage” to enable curators to identify those publications that will contribute most to the community’s accumulated knowledge. Although all MODs provide GO annotations to represent gene functions, many databases (especially those dedicated to multicellular eukaryotes, such as WormBase [28], FlyBase [29], and MGI [30]), must create, maintain, and use additional controlled vocabularies to comprehensively represent the biology of their target species (for example, covering such phenomena as anatomy and development). Despite differences in the scope, breadth, and depth of the data collected, all MODs share the broader goals of aggregating and presenting biological knowledge in a useful and readily understood format. They take common and, whenever possible, shared approaches to solving problems, ensuring consistency, and keeping pace with emerging trends in research and novel technologies adopted by their target communities. They also face identical biological, technical, and sociological challenges—most notably the growth of available data in a time of funding constraints that we address in this Comment article.

Rather than representing a drain on resources, biological databases in general, and MODs in particular, provide remarkably cost-effective support for biological research. MOD activities save time for individual laboratories, facilitate the interpretation of small- and large-scale experiments, and sustain diverse projects across many species. We believe that MODs will become increasingly critical to hypothesis generation, especially by those who aim to expand the scope of their research into novel areas, and a further reduction to MOD funding would lead to reductions in scientific innovation and commercial or therapeutic applications. Consistent with our views, an independent survey assessing the value of biological database services provided by the European Bioinformatics Institute (EBI) recently concluded that every £1 M spent on database funding yields £20 M in value to the global scientific community [31]. Further options for maintaining essential data resources should be explored. One is to share the financial burden more evenly between funders to reflect the geographical distribution of database users.

Taking into account all of the benefits provided by the model organism resources described here, we must challenge the suggestion that 0.37 % of the NIH biomedical research budget is a high price to pay to enable the thousands of research laboratories that depend upon these resources to function effectively or, indeed, exist. Reducing MOD funding might make modest short-term savings but would have potentially devastating long-term consequences. Motivating the scientific community to participate in the curation process is an important way of making funders’ dollars go further. Moreover, community involvement has benefits above and beyond any cost savings achieved. Experiments with model species remain essential to the biomedical research endeavor and the MODs that sustain such work deserve the active support of both those who fund them and those who use them.
